# Electrical Neuroimaging Reveals Timing of Attentional Control Activity in Human Brain

**DOI:** 10.1371/journal.pbio.0060081

**Published:** 2008-04-01

**Authors:** Jessica J Green, John J McDonald

**Affiliations:** Department of Psychology, Simon Fraser University, Burnaby, British Columbia, Canada; University of Oregon, United States of America

## Abstract

Voluntarily shifting attention to a location of the visual field improves the perception of events that occur there. Regions of frontal cortex are thought to provide the top-down control signal that initiates a shift of attention, but because of the temporal limitations of functional brain imaging, the timing and sequence of attentional-control operations remain unknown. We used a new analytical technique (beamformer spatial filtering) to reconstruct the anatomical sources of low-frequency brain waves in humans associated with attentional control across time. Following a signal to shift attention, control activity was seen in parietal cortex 100–200 ms before activity was seen in frontal cortex. Parietal cortex was then reactivated prior to anticipatory biasing of activity in occipital cortex. The magnitudes of early parietal activations were strongly predictive of the degree of attentional improvement in perceptual performance. These results show that parietal cortex, not frontal cortex, provides the initial signals to shift attention and indicate that top-down attentional control is not purely top down.

## Author Summary

To extract important details about objects in the environment, people must focus their attention on a specific location in space at any given moment. Research using functional magnetic resonance imaging (fMRI) has suggested that regions of the frontal and parietal lobes work together to control our ability to direct attention to a specific location in space in preparation for an expected visual object. However, the sluggishness of the hemodynamic response has made it difficult to obtain information from fMRI about the timing of activity. Electroencephalography (EEG) has provided information about the timing of neural activity, but the limitations of traditional source estimation techniques have made it difficult to obtain information about the precise location in the brain that the EEG signals are coming from. Thus, the sequence of activities within this frontal-parietal network remains unclear. We used a recently developed electrical neuroimaging technique—called beamforming—to localize the neural generators of low-frequency electroencephalographic (EEG) signals, which enabled us to determine both the location and temporal sequence of activations in the brain during shifts of visuospatial attention. Our results indicate that low-frequency signals in parietal cortex provide the initial signal to shift attention.

## Abstract 3

Localization of low-frequency brain rhythms reveals that within the frontal-parietal attentional control network, the parietal lobes provide the initial signal to shift attention in space.

## Introduction

Shifting attention to the expected location of an impending visual stimulus will improve the perception of that stimulus once it occurs there [1]. This perceptual improvement is considered to be a consequence of attentional-control operations that are performed by frontal and parietal regions of the human brain [2,3]. According to the widely accepted top-down model of voluntary attentional control, neural activities in frontal and parietal regions control the deployment of attention in space and eventually modulate the excitability of neurons in sensory-specific areas, which are responsible for processing of the upcoming stimulus. Traditionally, it has been assumed that the frontal lobes initiate top-down attentional control, because regions in frontal cortex are involved in the executive control of other cognitive and motor operations [3]. This assumption has been built into neural models of attentional control, in which one-way pathways from frontal cortex to parietal cortex to low-level visual areas subserve the voluntary control of spatial attention (Figure 1A) [2]. However, there is still much debate about the precise sequence of activity in the fronto-parietal network. Some evidence has suggested that frontal cortex becomes active before parietal cortex [4], while other evidence has suggested the opposite sequence [5,6]. This issue needs to be resolved in order to pin down the attentional control operations performed by the various regions in the network. For example, the latter sequence would suggest that parietal lobe is involved in the initiation of attentional control rather than the deployment or maintenance of attention in space, and thus necessitate a revision of current models of attentional control.

**Figure 1:**
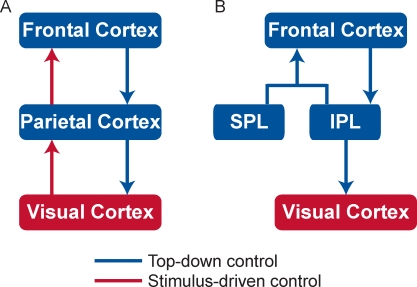
Models of Voluntary Attentional Control (A) Traditional model of top-down attentional control initiated in frontal cortex. (B) Model proposed in current study wherein attentional control is initiated by parietal structures.

A number of functional magnetic resonance imaging (fMRI) studies have confirmed the involvement of frontal and parietal lobes in the control of visual spatial attention [7–15], but the changes in blood flow that give rise to the fMRI signal are too sluggish to investigate the time courses of activities within these brain areas (however, attempts have been made to identify temporal order of activities using analytical techniques; see [16–18]). Advances in event-related fMRI have enabled researchers to separate attentional-control activity from subsequent attention effects on the neural responses to visual stimuli [11]. However, the hemodynamic response lasts for 10–20 s, whereas the neuro-cognitive operations involved in the control and deployment of attention in space each take only a fraction of a second [19]. Thus, the sequence of neural activations within the frontal-parietal network for attentional control cannot be elucidated with hemodynamic neuroimaging methods. By comparison, the scalp-recorded electroencephalogram (EEG) and event-related potentials (ERPs) triggered by sensory or cognitive processes reveal precisely the timing of brain activity associated with specific mental operations but traditionally have failed to provide precise information about the locations of active neurons.

In both ERP and fMRI studies, the neural correlates of attentional control are often investigated by examining the neural activity elicited by a symbolic cue (e.g., an arrow) that indicates which location to attend to in preparation for an upcoming target [20]. Typically, the neural responses between leftward-directing and rightward-directing cues are compared to one another to identify brain regions that are spatially selective for shifts of attention to particular locations [5,6,21–26]. Although this type of comparison has been useful for examining pre-target biasing in sensory areas, it has two important limitations with regards to identifying attentional control activity. First, not all of the spatially specific activities observed in the cue-target interval are related to attentional control. Some of these activities have been linked to low-level sensory responses elicited by the cue [14,24], motor preparation [21], saccadic suppression [23], and other nonattentional processes. Second, this method cannot detect any activity that is associated with shifts of attention to both left and right locations, because such spatially nonspecific activity is subtracted away. If, for example, activity in the right parietal lobe controls shifts of attention to both left and right visual fields [27], then that activity would go undetected.

To better isolate activity related to the control of attention shifts, researchers have begun to compare activity associated with the presentation of *attend* cues to activity associated with the presentation of *neutral* cues that either provide no information about the location of the impending target (i.e., noninformative cues) [6] or signify that the target will not occur (i.e., interpret cues) [4,14,28]. This method controls for the presentation of the sensory cue stimuli and also permits the detection of both spatially specific and spatially nonspecific neural responses. ERP and fMRI studies using this isolation method have provided converging evidence for bilateral activity in frontal and parietal regions of cortex [4,6,14]; but unfortunately, the sequence of attentional control activities in these regions has remained unclear.

One recent study that isolated attentional control with an interpret cue reported findings consistent with the top-down model of attentional control illustrated in Figure 1A using an fMRI-constrained dipole-modeling approach [4]. Neural sources of the grand-averaged attend-minus-interpret ERP difference waveforms were modeled with four dipoles placed at the coordinates of the bilateral frontal and parietal activations observed in a similar fMRI task [14]. The orientations of the dipoles were varied until the dipole model accounted for as much of the scalp-recorded ERP data as possible in the 400–1,900-ms time interval. The resulting fMRI-constrained model suggested that the left parietal source was active 200–300 ms after cue onset. Subsequent bilateral frontal source activity began 400 ms after cue onset and was sustained until target onset. Sustained bilateral activity was also seen in the parietal source waveforms beginning at 650 ms. Follow-up analyses suggested that the early left parietal source activity was not statistically significant; thus it was concluded that frontal cortex initiated attentional control about 400 ms after cue onset. However, the early parietal activity may have been obscured in three ways. First, the ERPs elicited by leftward and rightward directing cues were averaged together, thereby minimizing any spatially specific effects that might have occurred early in parietal cortex. Second, the analyses were not ideally designed to pick up small, transient ERP effects that may have occurred early in the cue-target interval. For example, differences between attend-cue ERPs and interpret-cue ERPs were analyzed statistically by measuring mean ERP amplitudes within consecutive 100-ms intervals that were not centered on any peaks in the attend-interpret difference waveforms. Moreover, the fMRI-constrained dipoles were not fit to the difference waveforms in the early (0–300 ms) portion of the cue-target interval. Third, the fMRI-constrained dipoles may have been at suboptimal locations to pick up any early activity in the parietal lobes.

Another study that isolated attentional control with a spatially noninformative cue reported findings that were inconsistent with the top-down model of attentional control illustrated in Figure 1A [6]. Bilateral activity was observed over frontal and parietal scalp sites, primarily at electrodes on the same side (ipsilateral) as the to-be-attended location, in the 300–450 ms time interval. This fronto-parietal activation was preceded by activity over the right parietal scalp at 250 ms, which suggests that right parietal cortex might initiate the sequence of attentional control. However, dipole source modeling of the isolated attentional control activity revealed sources in temporal, rather than parietal, cortices and was rejected as being physiologically implausible. Consequently, the neural sources of the early ERP activity seen over the parietal scalp remain unknown. In addition, some of the activities seen in the attend-neutral ERP difference waveforms may have reflected differences in overall arousal or motivation, because the attend cues and neutral cues were presented in separate tasks.

Given the results of the two studies that isolated attentional control with neutral cues, it is possible that parietal, rather than frontal, cortex initiates attentional control in the spatial cueing paradigm. To date, however, the methodological and analytical procedures used to investigate the sequence of attentional control in the fronto-parietal network have been insufficient to verify this hypothesis. Here we capitalized on recent advances in EEG source reconstruction to clarify the timing and sequence of activity related to attentional control. We examined event-related changes in EEGs recorded from 11 participants during an attention-cueing task [20], in which a cue presented at fixation indicated the likely location of an impending target (Figure 2A). This task enabled us to separate the neural activities associated with the cue-induced orienting of attention from the subsequent effects of attention on target processing. In addition, we included a subset of trials on which the cue provided no information about the location of the upcoming target. By comparing activity elicited by these noninformative (no-shift) cues with the activity elicited by the informative (shift) cues, we were able to isolate further the neural activities associated with attentional control from those associated with the sensory processing of the cue itself.

**Figure 2:**
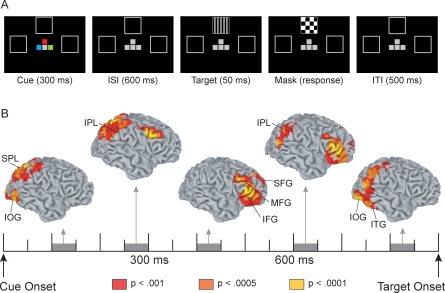
Stimuli and Results from the Main Experiment (A) Illustration of events on a single trial. ISI, inter-stimulus interval; ITI, inter-trial interval. (B) Spatio-temporal pattern of electrical brain activity (theta band) associated with top-down attentional control. Shown are surface-rendered maps of statistically significant increases in activity for a shift-up cue relative to the no-shift cue during five 50-ms time windows. Activity was distributed similarly in both hemispheres, so only the right-hemisphere activity is shown for simplicity. SPL, superior parietal lobe; IOG, inferior occipital gyrus; SFG, superior frontal gyrus; MFG, middle frontal gyrus; IFG, inferior frontal gyrus; IPL, inferior parietal cortex; ITG, inferior temporal gyrus.

We reconstructed the neural sources of EEG attentional control activity using a beamformer spatial filtering method [29,30]. The beamformer approach has several advantages over the dipole modeling approach. First, the beamformer method does not require a priori determination of the number of neural sources that may be giving rise to the scalp-recorded electrical fields. Second, the beamformer method outputs a volumetric image of neural activity throughout the brain, thereby facilitating the comparison of our results with those obtained from previous fMRI studies. Third, the beamformer method can be used to reconstruct neural sources of EEG in specific frequency bands. This enabled us to focus on oscillatory activity that we hypothesized would be important for visualizing attentional control activity across the entire cortex.

Prior studies have linked alpha band (8–14 Hz) and gamma band (>30 Hz) oscillations to attention and perception [31], but scalp-recorded oscillations in these frequency bands are primarily associated with the consequences of attention on activity in visual sensory areas [32–34] rather than the preceding attentional control operations in frontal and parietal cortices. To specifically examine attentional control activity, we opted to focus our beamformer analysis on the low-frequency theta band (4–7 Hz) oscillations. Although there is little or no existing evidence linking theta band activity to attention, we hypothesized that focusing on theta band oscillations would enable us to visualize attentional control activity across the cortex, because theta band oscillations have the following properties: (1) they reflect long-range communications between distant brain areas [35]; (2) they are carrier frequencies for high-frequency oscillations that reflect communications between nearby neurons (e.g., within a region) [36]; and (3) they have been previously linked to the working memory system [37], which is known to overlap with the spatial attention system [38]. To maximize our ability to home in on the attentional control areas that were identified in previous fMRI studies, we included both the evoked (phase-locked) and induced (non–phase-locked) activities in the analysis, because both would contribute to the hemodynamic response measured with fMRI.

We imaged neural sources of theta activity in each of 18 consecutive 50-ms intervals between cue and target. The reconstructed EEG source activities were then subjected to nonparametric statistical analyses [39] to determine which brain areas showed significant increases in activity associated with shifting attention. Based on previous electrophysiological studies, we made two predictions about the sequence of theta band activity during the voluntary control of visual attention. If voluntary attentional control is initiated in a completely top-down manner [4], activity would be seen first in frontal cortex, then in parietal cortex. Alternatively, if attentional control is initiated in parietal regions [5,6], activity should be seen first in parietal cortex and then in frontal cortex. Our results supported this latter hypothesis. Following a signal to shift attention, control activity was seen in parietal cortex 100–200 ms prior to activity in frontal cortex. Parietal cortex was then reactivated prior to anticipatory biasing of activity in occipital cortex.

## Results

Participants were most accurate to respond to targets that were validly cued (79%) and least accurate to respond to targets that were invalidly cued (69%), with intermediate accuracy for noninformatively cued targets (75%), *F* = 67.3, *p* < 0.0001. The location of the target neither influenced accuracy, *F* = 2.43, *p* = 0.12, nor interacted with cue validity, *F* = 1.14, *p* = 0.35. Follow-up comparisons revealed that accuracy for validly cued targets was significantly higher than for invalidly cued targets, *t* = 10.29, *p* < 0.00001, and for noninformatively cued targets, *t* = 6.86, *p* < 0.0001. Accuracy for noninformatively cued targets was also significantly higher than that for invalidly cued targets, *t* = 5.95, *p* = 0.0001. These behavioral results indicate that participants shifted their attention to the location indicated by the cue on shift trials and that target discrimination was improved when the cue accurately predicted the location of the upcoming target. A similar pattern of effects was observed for response times, with the shortest response times to validly cued targets, intermediate response times to noninformatively cued targets, and the longest response times to invalidly cued targets (674 ms, 714 ms, and 755 ms, respectively, *F* = 12.07, *p* = 0.002).

### Time Course of Attentional Control in Human Brain

Figure 2B displays surface-rendered maps of significant theta band activity for shift-up cues (relative to noninformative cues) in six representative time intervals. Activity associated with attentional control was observed in posterior brain areas during the first 300 ms following the appearance of the attention-directing cue. Initially, the activity was confined primarily to extrastriate regions of the occipital lobe, but by 200 ms, both the superior and inferior parietal lobes became active, and by 300 ms, the frontal lobes became active. Between 400 and 600 ms, the activity was confined to the inferior, middle, and superior frontal gyri. Following the activity in the frontal lobes, posterior parietal cortex became active for a second time (600–700 ms post-cue). During this second activation, activity was seen in the inferior, but not the superior, parietal lobule. This parietal activity was then followed by a second phase of activity in extrastriate visual cortex that extended along the middle and inferior occipital gyri into the inferior temporal lobes.

To better characterize the spatio-temporal sequences of neural activities involved in attentional control, we plotted the normalized power changes in theta band activity for the shift-up cue relative to the noninformative cue across the entire cue-target interval in occipital, parietal, and frontal regions of interest (ROIs) (Figure 3A). Activity in the inferior occipital gyrus (IOG) occurred in two phases, with an early peak at approximately 150–200 ms after the cue and a late phase that began approximately 600 ms after the cue and continued until the onset of the target stimulus. Activity in the inferior parietal lobule (IPL) showed a similar biphasic pattern. Notably, however, the first phase peaked later than in IOG, and the second phase peaked earlier. Activity in the superior parietal lobule (SPL) peaked early, around the same time as the initial peak activation in IPL, whereas activity in the middle frontal gyrus (MFG) peaked in the middle of the cue-target interval (300–600 ms post-cue). The sequence of peak activations across these ROIs suggests that an initial feed-forward sweep of activity sends information to executive control areas in frontal cortex, which then sends information back to lower areas.

**Figure 3:**
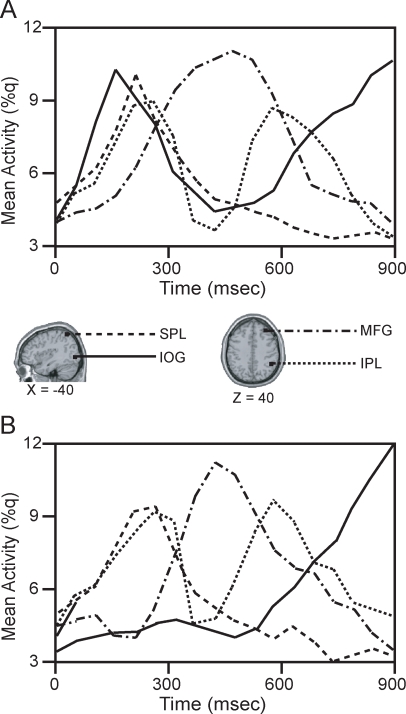
Time Courses of Activity in Occipital, Parietal, and Frontal ROIs (A) Activity elicited by multi-colored cues in the main experiment. (B) Activity elicited by letter cues in the follow-up experiment. Coordinates (*x*, *y*, *z*) of the ROIs were as follows: IOG: 43, −80, −1; SPL: 36, −58, 60; IPL: 49, −48, 44; MFGL: 34, 83, 44.

Similar patterns of attentional control activity were observed following shift-left and shift-right cues. In the case of shift-left and shift-right cues, however, some of the attention-related activity was lateralized (i.e., spatially specific). As shown in Figure 4, initial occipital activity following these cues was observed predominantly in the hemisphere contralateral to the to-be-attended location (i.e., the right hemisphere for shift-left cues and the left hemisphere for shift-right cues). The early activity in SPL was bilateral, whereas the early activity in IPL was greater in the hemisphere ipsilateral to the to-be-attended location than in the hemisphere contralateral to the to-be-attended location. Subsequent activations in MFG and occipital cortex were also larger in the ipsilateral hemisphere, whereas the late activity in IPL was bilateral.

**Figure 4:**
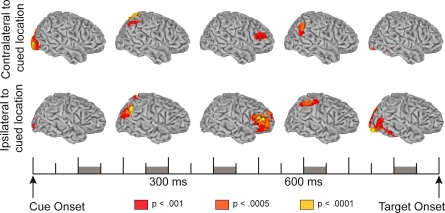
Statistically Significant Increases in Theta Band Activity for Shift-Left and Shift-Right Cues, Relative to Noninformative Cues The data for shift-left and shift-right have been collapsed such that the top row displays activity for brain regions contralateral to the cued location and the bottom row displays activity for brain regions ipsilateral to the cued location.

The early occipital and parietal activations are inconsistent with current models of top-down attentional control, according to which the signal to shift attention originates in frontal cortex [2]. Because our informative cues differed from the noninformative cue in one important respect—they contained a specific color that was known in advance to be predictive of target location—it is possible that the early activity was associated with attentive processing of the cues rather than control of attention shifts to the cued locations. To evaluate this possibility, we performed a follow-up experiment in which informative and noninformative cues did not differ on the basis of a simple feature. Letters were used to cue attention to the left, upper-middle, and right locations (L, U, and R, respectively) as well as for the non-informative cue (X). The results were almost identical to those obtained in the first experiment with the exception that no early occipital activity was observed (Figure 3B). This shows that the early occipital activity seen in the main experiment reflected attentional processing of the cue but that the early parietal activity reflected control of attentional shifts to the cued location.

### Control Activity Predicts Attentional Benefits in Performance

To determine whether the activations in occipital, parietal, and frontal regions led to modulation of perceptual processing of the subsequent target, we examined correlations between the activation magnitudes and the attention effects on target discrimination accuracy (Figure 5). All peak activations in the ROI time-courses correlated significantly with performance (*r*s > 0.78), except the initial activation in occipital cortex (Table 1). The lack of significant correlation with early occipital activity bolsters the conclusion that the early occipital activity reflected attentional processing of the informative cue itself. The significant correlations only at the peaks of activity in the ROIs provide compelling evidence that the early parietal activations as well as the later frontal, parietal, and occipital activations reflect attentional control operations that enhance processing of the impending visual target. Taken together, these peak activations accounted for 93% of the variability in attention effects on target discrimination accuracy (*R* = 0.97; *R^2^* = 0.93; *p* < 0.006). That is, the net activity within the attention-control areas identified here strongly predicts the level of attentional improvement in visual processing across participants.

**Figure 5:**
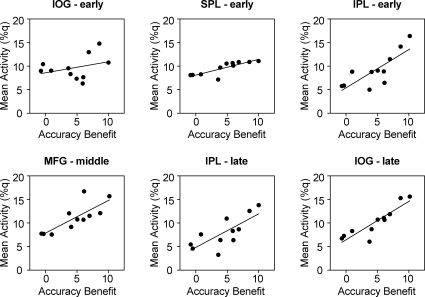
Correlations between the Peak Activations in the ROIs and the Attention-Cueing Effect on Target Discrimination Accuracy The accuracy benefit (*x*-axis) was computed by subtracting the accuracy for middle-upper targets following a noninformative cue from the accuracy for middle-upper targets following a shift-up cue.

**Table 1:**
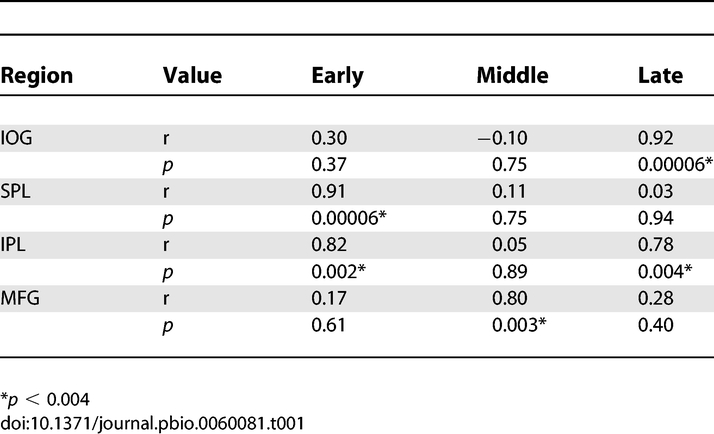
Correlations between Theta Band Activities in Regions of Attentional Control Network and Attentional Benefit on Target Discrimination Accuracy.

## Discussion

The present study used a recently developed technique for localizing the neural sources of scalp-recorded EEG to investigate the time course of brain activity associated with voluntary control of visuospatial shifts of attention. Although converging lines of evidence have pointed to the involvement of the frontal and parietal lobes in attentional control, the sequence of activity within the fronto-parietal control network has remained unclear due to the poor temporal resolution of fMRI and the limitations of ERP dipole source modeling. A number of alternatives have been proposed, including an entirely top-down system wherein shifts of attention are initiated by executive control regions of the frontal cortex [4] and a system wherein shifts of attention are initiated by activity in posterior brain regions that precedes frontal lobe activity [6]. Recent findings have provided support for the top-down model proposing that the frontal lobes initiate the sequence of attentional operations involved in the voluntary control of visuospatial attention shifts (Figure 1A). Our results, however, did not support this model. Instead, attentional-control activities in the parietal lobes were found to precede activity in the frontal lobes, which demonstrates that voluntary attentional control is not initiated solely by frontal cortex.

Given that IPL was active twice and SPL was active only early on, the two regions appear to mediate different attentional-control operations. The combined early activity in parietal cortex likely reflects a signal to switch attention to a specific location that is sent to executive control structures in frontal cortex. Recent neuroimaging studies indicate that activity in SPL is associated with shifting attention in spatial [40] and nonspatial [41] visual tasks, as well as in auditory and audiovisual tasks [42,43]. On this basis, we believe that SPL supplies the initial signal to switch attention, whereas IPL supplies spatial information about the to-be-attended location. The spatially nonspecific (bilateral) activation of SPL coupled with the spatially specific (predominantly ipsilateral) activation of IPL early in the cue-target interval following shift-left and shift-right cues supports this interpretation. The late activity in IPL may reflect operations involved in the marking of the to-be-attended location [9] or the actual deployment of attention to that location [11]. The late IPL activity was not sustained until target onset; thus, it is unlikely to reflect operations involved in maintenance of attention at the cued location.

The late activity in occipital and inferior temporal cortices began after the second phase of activity in IPL and was sustained until target onset. These areas are part of a ventral visual pathway that is involved in object processing and recognition [44]. Thus, the late occipito-temporal activity likely reflects anticipatory modulation of neuronal excitability in brain areas that would be responsible for processing sensory features of the upcoming target [45,46].

Following cues to shift attention to the nonlateralized location above fixation, attentional control activities in frontal and parietal areas as well as subsequent pre-target biasing in occipital cortex (relative to the noninformative cue) were largely bilateral. In contrast, attentional control activities in occipital, inferior parietal, and frontal cortices were lateralized following cues to shift attention to the left or right side of fixation. The spatially specific nature of the lateralized attentional control activity and subsequent pre-target biasing is in line with the lateralized organization of the primary visual pathways and is consistent with the observation of lateralized activity in ERP and fMRI studies examining activity following leftward and rightward-directing cues [4–6,11,14]. Increases in theta band activity were seen predominantly in cortical regions on the same side as the cued location, which suggests that this activity may be more closely associated with the anticipatory suppression of the to-be-ignored locations than the anticipatory enhancement of the to-be-attended location. The suppression of uncued locations has previously been linked to alpha band activity in this type of spatial cueing task [34]. The current results suggest that theta band activity also plays a role in the suppression of irrelevant information in order to maximize the attentional benefits for perception.

Our main finding—that voluntary attentional control is initiated in parietal cortex—is inconsistent with data from a recent combined ERP-fMRI study that reported initial activity in frontal cortex [4]. This discrepancy may be due to differences in the methods used to model brain activity. The electrical neuroimaging approach employed here used a spatial filtering technique unconstrained by any previous results or a priori hypotheses about the number of activated brain regions or the locations of the activated regions, whereas the conventional ERP-fMRI approach models electrical activity with a few discrete (dipolar) sources constrained to be at locations of fMRI activations. In addition, the beamformer technique enabled us to reconstruct the distributed neural sources of all oscillatory activity in the theta band, rather than just the evoked activity that is observed in the ERP. By comparison, the combined ERP-fMRI method faces the potential problem that induced changes in post-synaptic neural potentials are not seen in ERP waveforms (because they are not precisely phase-locked to events) but are likely associated with changes in hemodynamic responses. Such differences between the physiological contributions to ERP and fMRI signals may lead to errors in estimating the locations of ERP sources, which would, in turn, lead to errors in estimating the timing of ERP source activities. Unfortunately, the combined ERP-fMRI approach also eliminates the opportunity to use the fMRI data to evaluate the validity of the ERP source model, because data from the two methods are integrated.

We hypothesized that event-related changes in low-frequency theta band EEG oscillations would enable us to examine the spatial and temporal characteristics of activity in the voluntary attentional control network without any bias from previous fMRI results. To facilitate comparison of the present results with the results of recent fMRI studies of voluntary attentional control, we summarized the cortical sites of theta band activity across the entire cue-target interval in one image along with loci of fMRI activations [7–15]. This image, shown in Figure 6, reveals clusters of activations in occipital, parietal, and frontal regions of cortex. Although our use of standard head models, MRIs, and electrode positions likely limit our accuracy in identifying precisely the regions where attentional control activity took place, the loci of the frontal, parietal, and occipital theta band sources dovetail nicely with the foci observed in previous fMRI studies. In light of this converging evidence, it is clear that these frontal, parietal, and occipital regions play important roles in the control of spatial attention. In addition, these results provide evidence for a link between low-frequency theta band oscillations and attentional processes that heretofore has not been explored in the literature.

**Figure 6:**
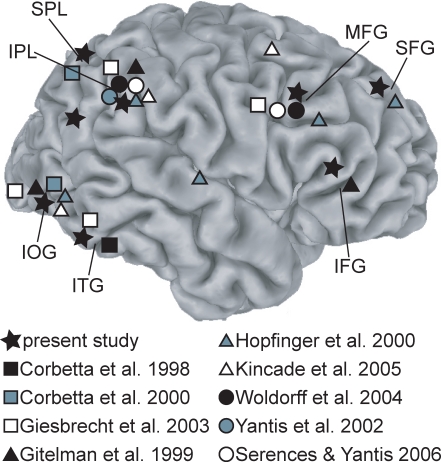
Summary of Results from Present Study and Previous Neuroimaging Studies of Attentional Control Symbols show approximate locations on the brain surface of activations based on the Talairach coordinates provided in each paper.

Our results show a clear link between low-frequency theta oscillations and attention. Prior studies have linked event-related changes in alpha and gamma band oscillations to attention and perception [32–34], but to date, theta band activity has been most closely associated with learning and memory [35–37]. Our focus on theta band activity was motivated by the hypothesis that theta band oscillations are critical for long-range communications between distant brain regions [35]. From this view, any cognitive operation that requires communication between distant brain regions should involve changes in theta-band activity. However, these low-frequency oscillations overlap in space and time with oscillations in many other frequency bands and are even coupled with high-frequency oscillations (e.g., high gamma [36]). Thus, it is unlikely that activity in any particular frequency band—such as theta, alpha, or gamma—is fully responsible for the many different attentional control operations performed by the fronto-parietal network. Other frequency bands may show different sequences of activities (i.e., frontal activity preceding parietal), and it remains to be seen how sequences of activity in different frequency bands relate to different attentional control processes. It is possible that event-related changes in specific frequency bands relate to specific attentional control operations performed by a given brain region, but it is also possible that the dynamics of attentional control activity across the cortex are more closely linked to coupling between different frequency bands (e.g., between theta and high gamma).

The electrical neuroimaging data provided here show that attentional control operations that follow the appearance of a symbolic spatial cue involve not just top-down signaling from frontal cortex but also an initial signaling from parietal cortex to indicate the need for an attention shift (Figure 1B). Moreover, the magnitude of the early parietal activity accurately predicted behavior on the subsequent perceptual task, indicating the importance of this early activity for accurate target identification. While it is possible that the attention system may be flexible and display different sequences of parietal and frontal activations with varying task requirements, it is clear that models of top-down control that posit a one-way passage of information from frontal to parietal cortex are insufficient to explain the complexities of voluntary attentional control.

## Methods

### 

#### Participants.

Twelve neurologically typical university students participated in the experiment after providing informed written consent. Data from one participant were not analyzed due to excessive blinking (on more than 30% of trials). Of the remaining 11 participants (9 female; mean age = 21.1 years), ten were right-handed and all had normal or corrected-to-normal vision. The experimental protocol was approved by the Simon Fraser University ethics committee.

#### Stimuli and procedures.

Stimuli were presented on a computer monitor in an unlit sound-attenuated chamber. Each experimental trial consisted of a 300-ms duration cue that either predicted the location (shift left, right, or up trials) of an impending target, or did not predict the target's location (non-informative cue/no-shift trials). After a 900-ms stimulus-onset asynchrony, five horizontally or vertically aligned bars appeared in one of the three target locations for 50 ms followed by a checkerboard pattern mask that remained on the screen until a response was made. Targets and masks were presented within landmark box outlines 2.5° in diameter that were present at the left, right, and upper-middle locations (each 8° from fixation) throughout the experimental blocks. The participants' task was to discriminate the orientation of the target bars and respond with a button press to indicate their choice. In the main experiment cues consisted of three colored squares, with a 1° radius from fixation. Each of the cue squares could be colored red (RGB = 255, 0, 0), green (RGB = 0, 225, 0), blue (RGB = 0, 204, 255), or violet (RGB = 204, 102, 204). For each participant, one color was predictive of the target location. The noninformative cue contained three nonpredictive colors. The letter cues used in the follow-up experiment were also 1° in height and 300-ms in duration (see [14] for similar cues). The letters were light gray in color (RGB = 201, 201, 201) and presented at fixation.

When the predictive cue color was present in the display, it accurately predicted the location of the impending target 80% of the time. On some trials (20%), the predictive color was not present (noninformative cue trials; the three cue squares were each one of the nonpredictive colors) and thus attention could not be directed to the probable location of the upcoming target in advance. To motivate participants to attend to the cued location whenever possible the task was designed to be difficult by adapting the luminance of the target bars on each trial to maintain an overall accuracy of 75%. Each participant performed 1,080 trials (288 trials in each of the shift-up, shift-left, and shift-right conditions; 216 trials in the no-shift condition). Participants' data were subjected to further analysis only if more than 70% of trials in each condition (i.e., more than 202 trials in each shift condition and more than 152 trials in the no-shift condition) were retained after blink and eye movement artifacts were removed.

#### Behavioral analysis.

The effect of attending to the cued location on ability to discriminate the subsequent target was assessed by comparing responses to targets when they were preceded by a valid cue (accurately predicting the target location), an invalid cue (predicting a location other than the target location), or a noninformative cue (not predictive of target location). The benefits of attending to the correct location were assessed by comparing responses validly cued and noninformatively cued targets, and the costs of attending to the incorrect location were assessed by comparing the responses to the invalidly cued and noninformatively cued targets. Both median response times and response accuracy were first entered into separate analyses of variance (ANOVAs) with factors for target location (left versus right versus up) and cue validity (valid versus invalid versus noninformative). Paired-samples *t*-tests were then performed to examine overall effects of attention on target processing (validly versus invalidly cued targets), as well as the benefits and costs of attending to the cued location.

#### Electrophysiological recording and analysis.

EEG was recorded from 63 tin electrodes referenced to the right mastoid. The horizontal electrooculogram was recorded bipolarly using two electrodes positioned lateral to the external canthi. Electrode impedances were kept below 10 kΩ. All signals were recorded with a bandpass of 0.1–100Hz (−3dB point; −12 dB per octave) and digitized at 500 Hz. Artifact rejection was performed to remove epochs that contained horizontal eye movements, detected on the horizontal electrooculogram channel, and blinks, detected at electrode FP1 positioned over the left eye.

Beamformer analysis was performed on each subject's data using the Multiple Source Beamformer implemented in the Brain Electrical Source Analysis software (BESA 5.1; Megis Software). The beamformer estimates the amount of activity that a source at a given point in the brain contributes to the activity observed at the scalp, while minimizing the contributions of sources at other points. By computing a separate beamformer for each point in a three-dimensional grid, this method yields spatially filtered estimates of activity at each point in the brain [29,30].

Each participant's EEG was transformed into the time-frequency domain using the complex demodulation technique implemented in BESA (see [47] for more details). The scalp signal is transformed into a complex signal, and the frequency of interest is shifted to 0 Hz then low-pass filtering with a Gaussian finite impulse response filter to remove other frequencies. The time-frequency sampling (1 Hz and 50 ms for frequencies between 2 and 20 Hz) indicates that the transform was repeated with each 1-Hz increment between 2 and 20 Hz as the frequency of interest, and the low-pass filter was applied in 50 ms increments throughout the epoch. This time-frequency sampling results in the signal being smoothed to a time-frequency signal with a full power width at half maximum of frequency ± 1.42 Hz and time ± 78.8 ms. Using a standard realistic head model, the beamformer source estimations for each shift condition (left, right, or up), normalized relative to activity in the non-informative condition, were output for each 2 × 2 × 2 mm voxel of the brain. The beamformer was applied to 18 separate 50-ms time windows that spanned the cue-target interval for the theta frequency band (4–7 Hz).

The output from the beamformer analysis for each time interval was subjected to a nonparametric statistical analysis using random permutation tests to determine activity significant across subjects [39]. The statistically significant activity (*p* < 0.001) was displayed on a surface rendered brain using fMRI analysis software [48]. ROIs (2 × 2 × 2 cm) were defined around the centroids of the activity seen in Figure 2B to determine the time course of activity within the main regions of interest. ROI analyses were performed using the raw averaged data, such that output values are the normalized power estimates (average *q* values) across subjects for that ROI.

To assess the degree to which neural activity during the cue-target interval was associated with subsequent perceptual enhancement of the target, response accuracy, and peak-activation magnitudes for the ROIs examined were subjected to a correlation analysis. Nonparametric correlations between the mean amplitude of activity in each ROI and the attentional benefit on target discrimination (accuracy for validly cued targets minus accuracy for non-informatively cued targets) were calculated at three different latencies during the cue-target interval. These latencies corresponded to the early peaks of activity (IOG = 150 ms, SPL = 200 ms, IPL = 250 ms, MFG = 250 ms), the mid-point of the cue-target interval (450 ms), and the late peaks of activity (IOG = 750 ms, SPL = 750 ms, IPL = 600 ms, MFG = 750 ms). The Type I error rate was kept below 0.05 by maintaining alpha = 0.004 for each of the 12 statistical tests. The ROI peaks that showed significant correlations with the accuracy benefits were then subjected to a multiple regression analysis to determine how well the combined activity in these regions predicted behavioral performance.

## Abbreviations

EEG: electroencephalogram
ERP: event-related potential
fMRI: functional magnetic resonance imaging
IOG: inferior occipital gyrus
IPL: inferior parietal lobe
MFG: middle frontal gyrus
ROI: region of interest
SPL: superior parietal lobe
